# Metabotyping of *Prunus sargentii*, *Prunus nipponica* var. *kurilensis*, and *Prunus maximowiczii* from Peter the Great Botanical Garden of BIN RAS

**DOI:** 10.3390/plants15101426

**Published:** 2026-05-07

**Authors:** Yuri G. Kalugin, Mayya P. Razgonova, Muhammad Amjad Nawaz, Kirill S. Golokhvast

**Affiliations:** 1 Komarov Botanical Institute of the Russian Academy of Sciences, Professor Popov Street, 2, 197022 Saint-Petersburg, Russia; 2 N.I. Vavilov All-Russian Institute of Plant Genetic Resources, B. Morskaya Street, 42-44, 190000 Saint-Petersburg, Russia; 3 Advanced Engineering School “Institute of Biotechnology, Bioengineering and Food Systems”, Far Eastern Federal University, Fr. Russian, Ajax, 10, 690922 Vladivostok, Russia; 4 Advanced Engineering School (Agrobiotek), National Research Tomsk State University, Lenin Ave., 36, 634050 Tomsk, Russia; 5 Laboratory for Research and Application of Supercritical Fluid Technologies in Agro-Food Biotechnology, National Research Tomsk State University, Lenin Ave., 36, 634050 Tomsk, Russia; 6 Siberian Federal Scientific Centre of Agrobiotechnology, Russian Academy of Sciences (RAS), 7 Centralnaya Street, 633501 Krasnoobsk, Russia

**Keywords:** *Prunus nipponica* var. *kurilensis*, *Prunus sargentii*, *Prunus maximowiczii*, polyphenols, Metabolomics, tandem mass spectrometry, Prunus

## Abstract

Species of the genus Prunus, including *Prunus nipponica* var. *kurilensis*, *Prunus sargentii*, and *Prunus maximowiczii*, are widely distributed in the Far Eastern region, covering the territories of Northern China, Korea, Japan, the Kuril Islands, Sakhalin Island, and Primorsky Region in Russia. As part of this study, the flowers of nine specimens of the aforementioned species were collected from the Peter the Great Botanical Garden of the Russian Academy of Sciences (RAS), which was founded in 1714 and is one of the oldest botanical gardens in Russia. This study is the first comprehensive metabolomic analysis of cherry blossoms from East Asia, with a particular focus on the varieties *P. nipponica* var. *kurilensis*, *P. sargentii* and *P. maximowiczii*. The main objective of the work was to identify and characterize biologically active polyphenolic substances and other chemotypes in the studied plant samples. Metabolomic analysis of flower extracts from three species of Prunus: *P. nipponica* var. *kurilensis*, *P. sargentii*, and *P. maximowiczii* revealed the presence of one hundred and eight polyphenol compounds and fourteen compounds belonging to other chemical groups. Principal component analysis showed that PC1 (26.6%) and PC2 (19.0%) explain 45.6% of the total variance. A clear separation of *P. maximowiczii* was observed, while *P. nipponica* from all regions was represented by a single species, and *P. sargentii* showed variability. Samples from Sakhalin were grouped separately. These results suggest that species identity and origin may influence the metabolic differentiation of the plant material studied. The observed separation of *P. maximowiczii* from other species may be due to both species-specific metabolism and adaptation to the environmental conditions in Sakhalin. A heatmap with hierarchical clustering revealed a clear clustering of samples based on their origin and species. Samples of *P. sargentii* from different sources were grouped together, indicating a similar metabolic profile. Samples of *P. nipponica* var. *kurilensis* formed a separate cluster with characteristic features of compound distribution. Samples of *P. maximowiczii* from Sakhalin also formed a separate cluster that was not related to the other two species. This supports the hypothesis that the subspecies that grow in the northern regions have a greater metabolic diversity. It is suggested that this richness of polyphenols is due to the harsh climatic conditions and the accompanying stress factors. The flowers of *P. nipponica* var. *kurilensis*, *P. sargentii*, and *P. maximowiczii* are characterized by a high content of biologically active compounds, which makes them promising objects for the creation of biologically active supplements and the development of new therapeutic agents in the pharmaceutical industry.

## 1. Introduction

The Peter the Great Botanical Garden at the V.L. Komarov Botanical Institute of the Russian Academy of Sciences (RAS) in St. Petersburg, Russia, is one of the largest botanical gardens in the world with such a northern location, located at the 59th parallel north. The history of plant testing on its territory dates back to 1714, when the Apothecary Garden was established for the medical office. Currently, the garden’s collection includes over 18,000 taxa, encompassing tropical and subtropical plants housed in a greenhouse complex covering approximately 1 hectare, as well as temperate plants grown in a 16.5-hectare arboretum park.

The main obstacle to growing plants from warmer regions in open ground, such as the iconic cherry blossom group (*Prunus* L.), is their lack of resistance to the local climate [1] and, above all, their poor winter hardiness, which is determined by their susceptibility to frost damage [2]. The practical value of these ornamental flowering plants primarily depends on the frequency of critical or abnormally harsh winters, which can lead to mass plant deaths or severe damage [3]. As relatively heat-loving exotics, sakuras could only survive in the open ground of the garden during the intervals between harsh winters.

One of the first species to appear in the open-field collection in 1915 was *Prunus maximowiczii* Rupr. (Miyamazakura) [4]. The duration of her trials is unknown, and repeated trials were conducted only after 1977. From 1950 to 1961, *Prunus sargentii* Rehder (Oyamazakura) and *Prunus nipponica* Matsum. var. *kurilensis* (Miyabe) E.H. Wilson (Takanazakura) was first introduced into open-field collections. While the most winter-hardy and vigorously growing of the Japanese cherries, *P. sargentii*, was introduced into world culture as early as 1890, *P. maximowiczii* in 1892, and *P. nipponica* var. *kurilensis* in 1915 [5].

Thus, the first tests of three types of cherries were conducted in St. Petersburg in the second half of the 20th century, when winter and summer temperatures were much colder than they are now. According to available meteorological data and phenological observations, there has been a noticeable trend towards climate warming since 1988, which has significantly intensified in the 21st century since 2006 [3]. The frequency of cold winters, which occurred approximately every 12 years in the 20th century, has decreased, and since the abnormally cold winter of 1986–1987, there have been no such winters with catastrophic consequences for woody exotics [3]. The freezing of the studied introductions has generally decreased, and in recent years, it has not been observed at all. The warming climate in St. Petersburg has also led to a significant increase in the length of the growing season, which is undoubtedly beneficial for plants. In the context of global warming and the concomitant shift in agro-climatic zones to the north and east, the phenomenon of climatic transformation becomes particularly important for the purposes of crop introduction. In most cases, this effect is perceived as positive, as increased temperatures create favorable conditions for the cultivation of a wider range of plant species in open fields compared to previous climatic periods [6]. Currently, 46 taxa of sakura, including six natural species, are grown on the territory of the park-arboretum. This collection, which includes species, hybrids, and cultivars, is a significant contribution to the study of cherry tree acclimatization in northern climates.

**Figure 1:**
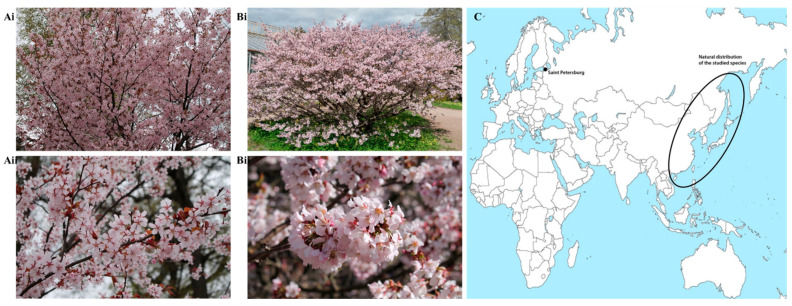
(**Ai**, **Aii**) *Prunus sargentii* Rehder (Photo by Yuri G. Kalugin, May 2024). (**Bi**, **Bii**) *Prunus nipponica* var. kurilensis (Miyabe) E.H. Wilson (Photo by Yuri G. Kalugin, May 2024). (**C**) A distribution area of *P. nipponica* var. kurilensis, *P. sargentii*, and *P. maximowiczii*.

The present study considers representatives of the genus Prunus, endemic to the Far East. Their distribution areas are visualized in Figure 1, which allows to clearly demonstrate the geographical distribution of these species.

The range of *P. maximowiczii* extends across the Russian Far East, China, Korea, and Japan. In its natural habitat, this species is a tree that grows to a height of 15–20 m and a diameter of 30 cm. It blooms after the leaves have fully opened. The flowers are white, 10–16 mm in diameter, and are arranged in clusters of 3–9. The drupes are small, about 8 mm in diameter, black, with a purple-black juice, and are bitter and inedible. This species is promising for landscaping, both in single and group plantings, as it is decorative during flowering and has a vibrant autumn foliage color. In the Arboretum of the Botanical Institute of the RAS, it is represented by three specimens. One is grown from seeds obtained from the island of Sakhalin, sprouted in 1977; the second was brought in 1898 by a 2-year-old plant from Primorsky Territory (Russian Far East); the third is a self-seeding plant from the scientific experimental station Otradnoye (120 km north of St. Petersburg), obtained in 2008 at the age of 3 years.

The Kuril variety of the Nippon cherry (*P. nipponica* var. *kurilensis*) comes from the island part of the Russian Far East and Japan. In nature, it is a shrubby tree up to 6 m tall, with large white or pale pink flowers up to 2.5 cm in diameter. The fruits are spherical, 8–10 mm in diameter, and black. It is very decorative during flowering and has potential for expanding its cultural range. Three specimens are grown in the arboretum of the Biological Institute of the RAS, with the oldest specimen being 80 years old. Another specimen is a vegetative offspring of the first, propagated by cuttings in 1978.

Oyamazakura, or *P. sargentii*, is distributed in the Russian Far East (Primorsky Territory, South Sakhalin, Kuril Islands), as well as in China, Korea and Japan. The tree reaches larger sizes than the Maximowicz cherry, up to 20 m in height and 40 cm in trunk diameter. The flowers are pale pink, up to 35 mm in diameter, and are borne singly or in clusters of one to three. They bloom at the same time as the leaves, which are dark-colored, bronze, or purple at this time. The fruits are purple-black, spherical, and 8 to 10 mm in diameter, and are considered inedible. In the Arboretum of the Botanical Institute of the RAS, the most represented species of sakura is represented by more than 100 specimens, with the oldest specimen being a plant that was brought to the arboretum in 1984 from Sakhalin.

The results of the metabolomic study presented in the publication refer to three iconic cherry species grown in the garden in open ground: *P. maximowiczii*, *P. nipponica* var. *kurilensis* and *P. sargentii* Rehder (Oyamazakura). These species, selected for their adaptive potential and ornamental value, are being studied to understand their metabolic responses to the unique environmental conditions of the garden.

Yoshinari et al. conducted an exhaustive investigation into the phytochemical composition of the bark of *Prunus jamasakura*, focusing on the isolation and characterization of flavonoids and lignans. Through rigorous spectroscopic analysis, three novel compounds were identified: a flavanone xyloside, a sesquilignan, and a neolignan. These compounds were elucidated alongside several previously known constituents, thereby enriching the understanding of the bark’s chemical profile. The structural elucidation of these components was achieved through a combination of NMR, MS, and other advanced spectroscopic techniques, ensuring the accuracy and reliability of the results [7].

A comprehensive investigation was conducted to assess the coumarin content in leaves and bark of various *Prunus* species. The study revealed the presence of herniarin in the leaves of all three *Prunus* species previously documented to contain this compound (*P. mahaleb*, *P. pensylvanica*, *P. maximowiczii*). However, the bark of *P. maximowiczii* was found to contain scopolin. Notably, herniarin was also detected in the leaves of *P. maackii* and two cultivars of *P. cerasus* (Montmorency and Northstar), while the bark of these species exclusively harbored scopolin. Scopolin, and occasionally scopoletin, was identified in the bark of *P. cyclamina*, *P. nipponica* var. *kurilensis*, *P. serotina*, *P. verecunda*, *P. virginiana*, and three grafted cultivars of *P. avium* (‘Bing’, ‘Black Tartarian’, ‘Lambert’). This research underscores the genetic and phenotypic variability of coumarin biosynthesis within the *Prunus* genus, highlighting the potential for further exploration into the ecological and evolutionary implications of these compounds [8].

The wood of *Prunus tomentosa* is characterized by the presence of d-catechin and a coumarin glucoside tomenin, which constitute its primary chemical constituents. This species is classified within the section Microcerasus of the subgenus Cerasus, a taxonomic division that has been further subdivided into four sections: Pseudocerasus, Eucerasus, Phyllomahaleb, and Microcerasus. Woods derived from species belonging to the sections Pseudocerasus, Eucerasus, and Phyllomahaleb are known to accumulate flavanones and flavones. The analysis of Prunus woods provides evidence suggesting that the biosynthesis of catechin and leucoanthocyanin does not parallel that of flavanones and flavones. This implies that catechins and the group of flavanones and flavones might be synthesized through distinct biosynthetic pathways. The wood of Prunus tomentosa exemplifies this divergence, as it exclusively produces d-catechin, while neither flavanones nor flavones are synthesized. Instead, the species synthesizes a coumarin glycoside, which is hypothesized to be a direct derivative of cinnamic acid. The production of tomenin and d-catechin in Prunus tomentosa serves as a compelling example supporting the notion that these compounds are derived from different biosynthetic pathways [9].

In this study, we report the metabolome composition of *P. nipponica* var. *kurilensis*, *P. sargentii*, and *P. maximowiczii* varieties’ flower extracts from nine varieties separated by growing *Prunus* in the Peter the Great Botanical Garden using HPLC-ESI-MS and ESI-MS/MS analyses.

## 2. Results

### Global Metabolome Profiles of P. nipponica var. kurilensis, P. sargentii, P. maximowiczii

The HPLC conditions were optimized to obtain maximal resolution and signal within a minimal run time. Various chromatographic conditions, such as mobile phase composition, injection volume, flow rate, column temperature, and gradient program, were studied and optimized for the separation of polyphenol compounds. Different mobile phase compositions (ethanol–water, ethanol-0,1% (*v*/*v*) formic acid aqueous solution, acetonitrile–water, and acetonitrile –0.1% (*v*/*v*) formic acid aqueous solution) were tested in the gradient program at 0.25 mL/flow rate. A mobile phase composed of 0.1% (*v*/*v*) formic acid aqueous solution (A) and acetonitrile (B) at a 0.25 mL/min flow rate and 17 °C column temperature was found optimal for the resolution of the maximum number of peaks in extracts of *P. nipponica* var. *kurilensis*, *P. sargentii*, and *P. maximowiczii* within 60 min.

We were able to identify one hundred twenty-two chemical compounds from extracts of *P. nipponica* var. *kurilensis*, *P. sargentii*, and *P. maximowiczii*: one hundred eight chemical compounds from the polyphenol group and fourteen chemical compounds from other chemical groups. All the identified polyphenols and other compounds, along with molecular formulas, and MS/MS data for *P. nipponica* var. *kurilensis*, *P. sargentii*, and *P. maximowiczii* are summarized in Appendix A Table A1. The polyphenols detected in our study were further categorized as flavan-3-ols, flavones, flavonols, tannins, anthocyanidins, phenolic acids, lignans, coumarins, stilbenes, etc. Overall, the metabolites detected in our study belonged to 20 compound classes. The highest number of metabolites was flavonols (38), followed by phenolic acids (22), flavones (15), anthocyanins (15), and flavan-3-ols (8). These numbers indicate that *P. nipponica* var. *kurilensis*, *P. sargentii*, and *P. maximowiczii* flowers are rich in polyphenol content.

**Figure 2:**
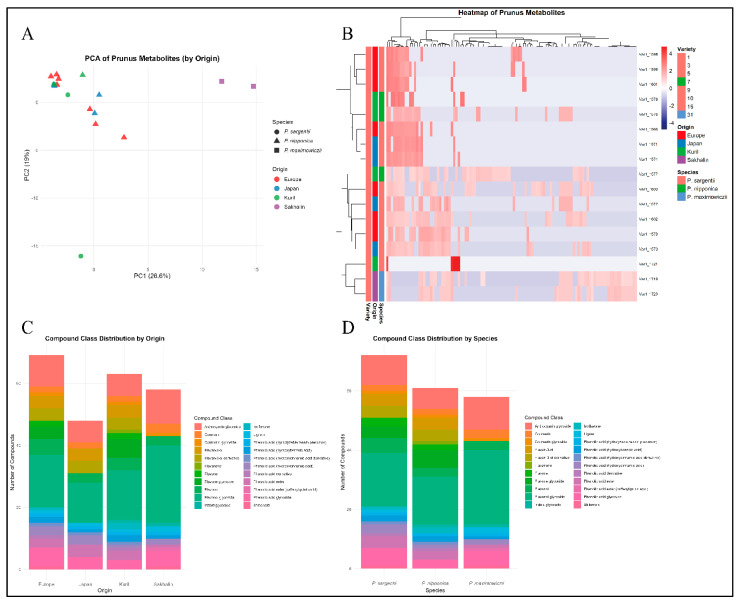
Global metabolome profiles of *P. nipponica* var. *kurilensis*, *P. sargentii*, and *P. maximowiczii*. (**A**) Principal component analysis, (**B**) heatmap, (**C**,**D**) compound counts by origin and species.

Principal component analysis showed that PC1 (26.6%) and PC2 (19.0%) explained 45.6% of the total variance. Clear separation of *P. maximowiczii* was observed, whereas *P. nipponica* was observed from all origins. However, *P. sargentii* exhibited variation. From the point of origin, the samples from Sakhalin were grouped separately. These results suggest that species identity and origin may contribute towards metabolic differentiation in the studied plant material (Figure 2A). Additionally, the small number of samples from each geography and for each species (especially for *P. maximowiczii*), and the confounding of the species with origin, need cautious interpretation. The observed separation of *P. maximowiczii* from other species may reflect either species-specific metabolism or adaptation to Sakhalin environmental conditions. Heatmap with hierarchical clustering revealed clear clustering of samples primarily by origin as well as species. The *P. sargentii* samples representing multiple origins clustered together, indicating a shared metabolite profile. *P. nipponica* var. *kurilensis* samples formed a separate cluster characterized by distinct compound abundance patterns. *P. maximowiczii* samples from Sakhalin also formed a distinct cluster, separate from the other two species (Figure 2B).

Among the compound classes, flavonol glycosides were the most representative group, followed by anthocyanin glycosides, flavonols, phenolic acid glycosides, and others. Samples from Sakhalin had the highest flavonol glycosides, followed by Europe, Kuril, and Japan. *P. maximowiczii* was rich in flavonol glycosides, anthocyanin glycosides, phenolic acid glycosides, flavonols, coumarins, and others. *P. sargentii* had a higher number of flavonols, phenolic acid glycosides, flavone glycosides, phenolic acid esters, together with flavonol glycosides and anthocyanin glycosides (Figure 2C,D).

**Figure 3:**
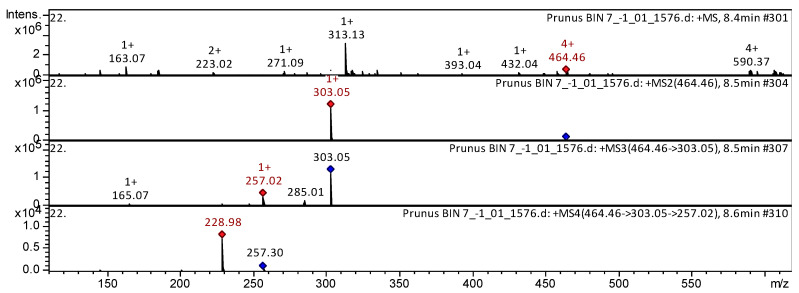
CID-spectrum of quercetin 3-*O*-glucoside from extracts of *P. nipponica* var. *kurilensis*, *m*/*z* 285.35. At the top is an MS scan in the range of 100–1700 *m*/*z*; at the bottom are fragmentation spectra (from top to bottom): MS2 of the protonated quercetin 3-*O*-glucoside ion (464.46 *m*/*z*, red diamond), MS3 of the fragment 464.46→303.05 *m*/*z*, and MS4 of the fragment 464.46→303.05→257.02 *m*/*z*.

**Figure 4:**
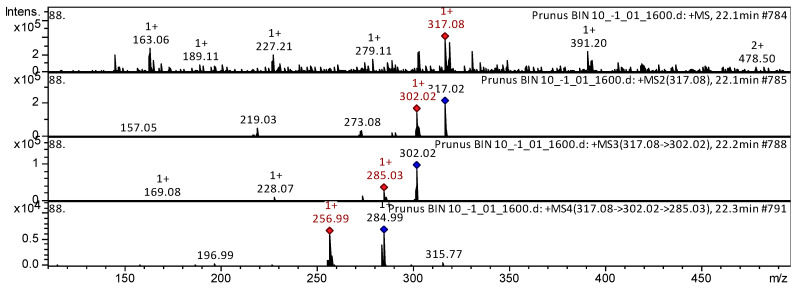
CID-spectrum of isorhamnetin from extracts of *P. sargentii*, *m*/*z* 317.08. At the top is an MS scan in the range of 100–1700 *m*/*z*; at the bottom are fragmentation spectra (from top to bottom): MS2 of the protonated isorhamnetin ion (317.08 *m*/*z*, red diamond), MS3 of the fragment 317.08→302.02 *m*/*z*, and MS4 of the fragment 317.08→302.02→285.03 *m*/*z*.

**Figure 5:**
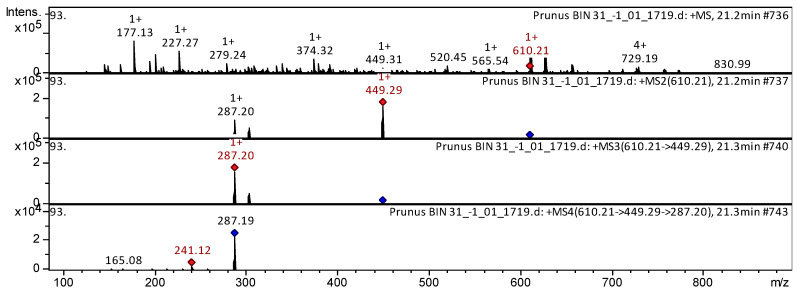
CID-spectrum of kaempferol 3,7-*O*-diglucoside from extracts of *P. maximowiczii*, *m*/*z* 610.21. At the top is an MS scan in the range of 100–1700 *m*/*z*; at the bottom are fragmentation spectra (from top to bottom): MS2 of the protonated kaempferol 3,7-*O*-diglucoside ion (610.21 *m*/*z*, red diamond), MS3 of the fragment 610.21→449.28 *m*/*z*, and MS4 of the fragment 610.21→449.28→287.20 *m*/*z*.

As a result of our mass spectrometric research, the most widely represented polyphenolic group of compounds was identified as flavanols (38). Three tentatively identified CID-spectra (collision-induced spectrum) of flavanols in *P. nipponica* var. *kurilensis*, *P. sargentii*, and *P. maximowiczii* extracts are presented below (Figure 3, Figure 4 and Figure 5). Flavanol quercetin 3-O-glucoside was found in the extracts from flowers of *P. nipponica* var. *kurilensis*, *P. sargentii*, and *P. maximowiczii* (Figure 3).

The mass spectrum in positive ion mode of quercetin 3-O-glucoside from extracts of the flowers from *P. nipponica* var. *kurilensis* is shown in Figure 3. The [M + H]^+^ ion produced one fragment ion at *m*/*z* 303.05. The fragment ion with *m*/*z* 303.05 yielded three daughter ions at *m*/*z* 285.01, *m*/*z* 257.02, and *m*/*z* 165.07. The daughter ion produced one fragment ion at *m*/*z* 228.98. This bioactive substance was identified in mass spectrometric studies as quercetin 3-O-glucoside in extracts of *Rubus occidentalis* [10], *Ribes meyeri* [11], *Lonicera henryi* [12], *Lonicera japonica* [13], Andean blueberry [14], *Rhus coriaria* [15], Mexican lupine species [16], *Fagopyrum esculentum* [17], Embelia [18], *Aspalathus linearis* [19], *Rosa acicularis*, *Rosa rugosa*, and *Rosa amblyotis* [20].

Flavanol isorhamnetin was found in the extracts from flowers of *P. nipponica* var. *kurilensis*, *P. sargentii*, and *P. maximowiczii* (Figure 4).

The mass spectrum in positive ion mode of isorhamnetin from extracts of the flowers from *P. nipponica* var. *kurilensis* is shown in Figure 4. The [M + H]^+^ ion produced four fragment ions at *m*/*z* 302.02, *m*/*z* 273.08, *m*/*z* 219.03, and *m*/*z* 157.05. The fragment ion with *m*/*z* 302.02 yielded three daughter ions at *m*/*z* 285.03, *m*/*z* 228.07, and *m*/*z* 169.08. The daughter ion with *m*/*z* 285.03 produced two fragment ions at *m*/*z* 256.99 and *m*/*z* 196.99. This bioactive substance was identified in mass spectrometric studies as isorhamnetin in extracts of *Inula viscosa* [21], Andean blueberry [14], eucalyptus [22], *Astragali Radix* [23], *Stevia rebaudiana* [24], Embelia [18], *Rosmarinus officinalis* [25], propolis [26], *Rosa acicularis*, *Rosa rugosa*, *Rosa amblyotis* [20], and *Phoenix dactylifera* [27].

Flavonol kaempferol 3,7-*O*-diglucoside was found in the extracts from flowers of *P. maximowiczii* (Figure 5).

The mass spectrum in positive ion mode of kaempferol 3,7-*O*-diglucoside from extracts of the flowers of *P. maximowiczii* is shown in Figure 5. The [M + H]^+^ ion produced two fragment ions at *m*/*z* 449.29 and *m*/*z* 287.20. The fragment ion with *m*/*z* 449.29 yielded one daughter ion at *m*/*z* 287.20. The daughter ion with *m*/*z* 287.20 produced two fragment ions at *m*/*z* 241.12 and *m*/*z* 165.08. This phenolic compound was identified in mass spectrometric studies as kaempferol 3,7-*O*-diglucoside in extracts of Rapeseed petals [28], *Taraxacum officinale* [29], *Rosemary*, *Mint*, *Salvia*, *Thymus vulgaris*, and *Laurus* [30].

**Figure 6:**
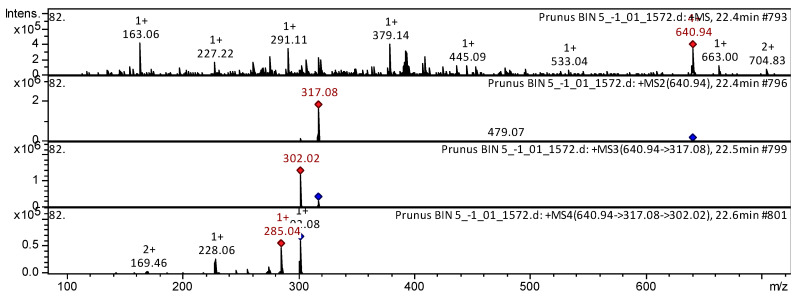
CID-spectrum of petunidin 3,5-di-*O-beta-D*-glucoside from extracts of *P. sargentii*, *m*/*z* 640.94. At the top is an MS scan in the range of 100–1700 *m*/*z*; at the bottom are fragmentation spectra (from top to bottom): MS2 of the protonated petunidin 3,5-di-*O-beta-D*-glucoside ion (640.94 *m*/*z*, red diamond), MS3 of the fragment 640.94→317.08 *m*/*z*, and MS4 of the fragment 640.94→317.08→302.02 *m*/*z*.

Anthocyanins are major color-forming pigments in the flowers of *P. nipponica* var. *kurilensis*, *P. sargentii*, and *P. maximowiczii*. The extract analyses of flowers of *Prunus* (four varieties) by employing HPLC-ESI-MS and ESI-MS/MS resulted in the detection of seventeen anthocyanins. A notable result was that the extracts of the flowers of *P. nipponica* var. *kurilensis*, *P. sargentii*, and *P. maximowiczii* varieties contained delphinidin 3-*O*-(6-*O-p*-coumaroyl) glucoside, petunidin 3-*O*-(6″-caffeoyl) hexoside, delphinidin-3,5-diglucoside, malvidin 3-*O*-(6″-*O-p*-coumaroyl) glucoside, petunidin 3-*O*-glucoside-5-*O*-glucoside, cyanidin 3-*O*-rutinoside, etc. Below in the article, several mass spectra of the most frequently occurring anthocyanins in all four *Prunus* varieties are presented as examples. Anthocyanin petunidin 3,5-di-*O-beta-D*-glucoside was found in the extracts from flowers of *P. sargentii* (Figure 6).

The mass spectrum in positive ion mode of petunidin 3,5-di-*O-beta-D*-glucoside from extracts of the flowers from *P. sargentii* is shown in Figure 6. The [M + H]^+^ ion produced one fragment ion at *m*/*z* 317.08. The fragment ion with *m*/*z* 317.08 yielded one daughter ion at *m*/*z* 302.02. The daughter ion with *m*/*z* 302.02 produced three fragment ions at *m*/*z* 285.04, *m*/*z* 228.06, and *m*/*z* 169.46. This anthocyanin was identified in mass spectrometric studies as petunidin 3,5-di-*O-beta-D*-glucoside in extracts of *Grape* [31], *Vitis vinifera* [32], *Vitis labrusca* [33], and *Loropetalum chinense* [34].

**Figure 7:**
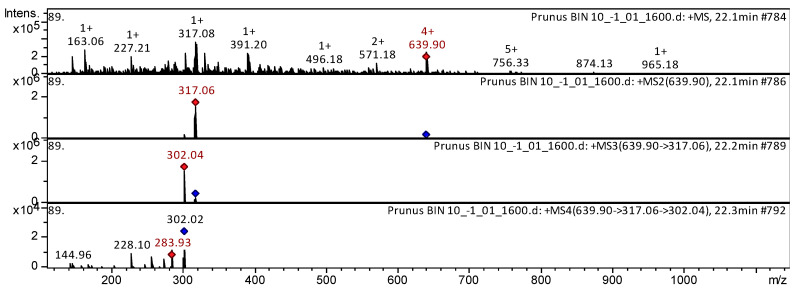
CID-spectrum of malvidin 3-*O*-(6″-*O-p*-coumaroyl)hexoside from extracts of *P. sargentii*, *m*/*z* 639.90. At the top is an MS scan in the range of 100–1700 *m*/*z*; at the bottom are fragmentation spectra (from top to bottom): MS2 of the protonated malvidin 3-*O*-(6″-*O*-p-coumaroyl)hexoside ion (639.90 *m*/*z*, red diamond), MS3 of the fragment 639.90→317.06 *m*/*z*, and MS4 of the fragment 639.90→317.06→302.04 *m*/*z*.

Anthocyanin malvidin 3-O-(6″-O-p-coumaroyl)hexoside was found in the extracts from flowers of *P. sargentii* (Figure 7).

The mass spectrum in positive ion mode of malvidin 3-*O*-(6″-*O-p*-coumaroyl)hexoside from extracts of the flowers from *P. sargentii* is shown in Figure 7. The [M + H]^+^ ion produced one fragment ion at *m*/*z* 317.06. The fragment ion with *m*/*z* 317.08 yielded one daughter ion at *m*/*z* 302.04. The daughter ion with *m*/*z* 302.04 produced three fragment ions at *m*/*z* 283.93, *m*/*z* 228.10, and *m*/*z* 144.96. This anthocyanin was identified in mass spectrometric studies as malvidin 3-O-(6″-O-p-coumaroyl)hexoside in extracts of grapevine varieties [35], *Vitis vinifera* [36].

## 3. Discussion

**Figure 8:**
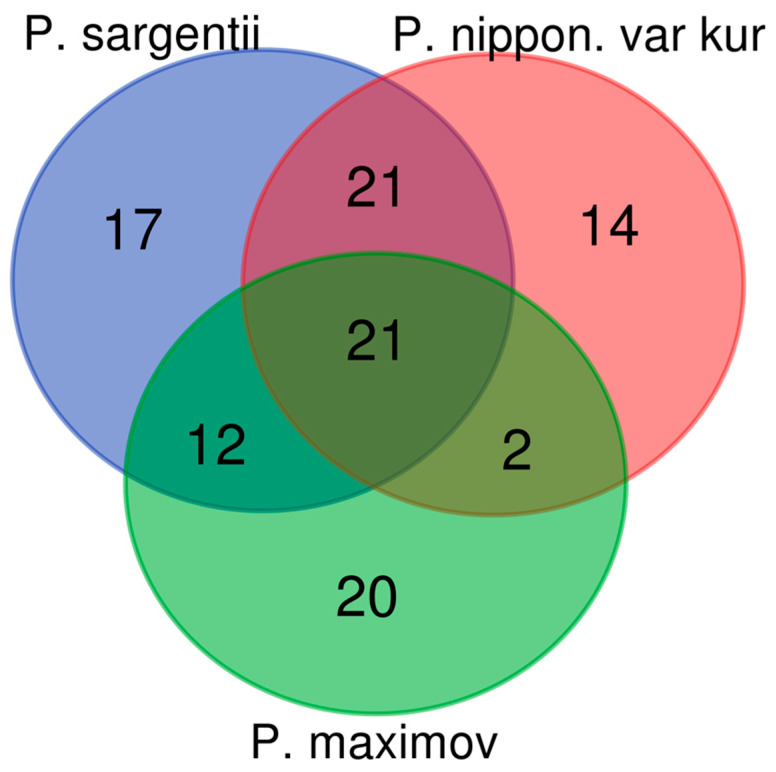
The Venn diagram shows the similarities and differences between varieties *P. nipponica* var. *kurilensis*, *P. sargentii*, and *P. maximowiczii.*

The Venn diagram below demonstrates the intersection and differentiation of polyphenolic complexes in three species of the genus Prunus (Figure 8).

Figure 8 shows the similarities and differences between varieties *P. nipponica* var. *kurilensis*, *P. sargentii*, and *P. maximowiczii*. The following Venn diagram elucidates the compositional similarities and differences within the polyphenolic complexes of extracts derived from the flowers of three distinct species: *P. sargentii*, *P. nipponica* var. *kurilensis*, and *P. maximowiczii*. The diagram meticulously illustrates the maximum congruence in identified polyphenolic constituents between *P. sargentii* and *P. nipponica* var. *kurilensis* (43.5%). In contrast, a markedly lower degree of overlap in polyphenolic composition was discerned between *P. sargentii* and *P. maximowiczii* (30.6%), as well as between *P. nipponica* var. *kurilensis* and *P. maximowiczii* (24.1%).

The Jaccard index was used to identify similarities and differences in the polyphenolic components of the three species of the genus Prunus (Table 1). This statistical tool, also known as the Jaccard similarity coefficient, is widely used in ecology and biostatistics to assess the degree of similarity and diversity between sets of samples [37]. The results of the analysis showed that the maximum degree of similarity is observed between the species *P. nipponica* var. *kurilensis* and *P. sargentii*, amounting to 0.5393.

The following is Table 2 showing the distribution of the polyphenol compounds in *P. nipponica* var. *kurilensis*, *P. sargentii*, and *P. maximowiczii.*

During mass spectrometric studies of the extracts, a large number of polyphenolic compounds were identified that had not previously been mentioned by other authors in extracts of the species *P. sargentii*, *P. nipponica* var. *kurilensis*, and *P. maximowiczii.* These are the following chemical compounds: Isorhamnetin 3-O-(6″-O-rhamnosyl-hexoside); 2,3,4,5-Tetrahydroxybenzoic acid; Acacetin O-glucoside; Luteolin-O-hexoside; Gengkwanin; Petunidin 3-O-p-coymaroyl hexoside; Cirsiliol; Methyl ellagic acid valerylpentose; Acacetin 7-O-glucoside; Petunidin 3-O-(6″-caffeoyl)hexoside; Dihydroxy-dimethoxy(iso)flavone; Luteolin 7-O-glucoside; Isorhamnetin-3-O-rutinoside; Calycosin; Petunidin-3-rutinoside; Isorhamnetin 3-O-neohesperidoside; Isorhamnetin O-rhamnosyl-hexoside; Kaempferol 3,7-dirhamnoside; Delphinidin-3,5-diglucoside; Delphinidin-3-caffeoylglucoside; Quercetin-O-dihexoside; Vimalin; Cyanidin O-deoxyhexoside; Ellagic acid-rhamnoside; Matairesinol; Kaempferol 3-O-pentoside; Luteolin 7-O [6-dihydrogalloyl]-glucosyl-8-C-Pentosyl-(1-6) glucoside; Quercetin O-(caffeyl)hexoside; Kaempferol-3,7-O-diglucoside; Quercetin 3-O-pentoside; Afzelin; Rhamnocitrin; Avicularin; Quercetin-3-O-arabinoside; Chlorogenic acid; Ellagic acid-O-deoxyhexoside; Quercetin-3-alpha-xylopyranoside; Scopoletin; Resveratrol.

It is noteworthy that a significant portion of the aforementioned compounds exhibit high biological activity, positioning them as promising candidates for both nutritional supplements and therapeutic agents. These multifaceted potentials underscore their utility in the design and optimization of novel dosage forms and advanced treatment strategies.

For example, the pharmacological properties of scopoletin have been extensively investigated, revealing a multifaceted spectrum of biological activities including potent antioxidant, antidiabetic, hepatoprotective, neuroprotective, and antimicrobial effects. These activities are mediated through intricate intracellular signaling mechanisms, which underscore the compound’s potential as a therapeutically valuable natural product. Notably, scopoletin exhibits significant anti-inflammatory and antitumor activities, which are intricately linked to a network of signaling pathways. These pathways include the nuclear erythroid factor 2-related factor 2 (Nrf2) pathway, the apoptosis/p53 signaling cascade, the nuclear factor-κB (NF-κB) pathway, the autophagy signaling axis, the hypoxia-inducible factor (HIF) pathway, the signal transducer and activator of transcription 3 (STAT3) pathway, the Wnt-β pathway, and the Notch signaling pathway. A comprehensive elucidation of the key molecular targets within these signaling networks holds promise for enhancing our understanding of scopoletin’s therapeutic potential, positioning it as a promising bioactive compound for the development of novel treatments for various diseases [38].

Or another vivid example: biologically active properties of flavone genkwanin. In vitro and in vivo morphological and pharmacological investigations have conclusively demonstrated that genkwanin displays exceptional antioxidant and anti-inflammatory properties. Furthermore, genkwanin exhibits antihyperglycemic activity by activating glucokinase, which holds significant potential for mitigating metabolic syndrome and diabetes. Genkwanin also possesses cardioprotective and neuroprotective effects, thereby reducing the risk of cardiovascular diseases. Additionally, genkwanin exhibits antitumor activity, antibacterial, antiviral, and dermatoprotective properties. These beneficial effects are mediated through intricate subcellular, cellular, and molecular mechanisms, including the induction of apoptosis and the inhibition of cancer cell growth and proliferation [39].

Another interesting example is the biological activity of the flavone herbacetin. Herbacetin exhibits potent inhibition of cardiomyocyte hypertrophy both in vitro and in vivo, attenuating the production of reactive oxygen species (ROS) and calcium dyshomeostasis. Furthermore, it modulates the phosphorylation status of SGK1, thereby regulating its downstream signaling cascade, including the transcription factor forkhead box O1 (FoxO1). Collectively, these findings suggest that a class of flavonoids structurally analogous to herbacetin holds significant promise as novel candidates for the development of therapeutics targeting cardiac hypertrophy [40].

The flavone compound cirsiliol demonstrates potent anti-proliferative effects against osteosarcoma cells, inducing apoptosis and downregulating AKT phosphorylation. Consequently, cirsiliol enhances the expression of FOXO1, a transcription factor with established roles in cell cycle arrest and apoptosis. These findings suggest that cirsiliol holds significant promise as a novel therapeutic agent for osteosarcoma treatment, warranting further investigation into its molecular mechanisms and potential clinical applications [41].

Kaempferitrin (kaempferol 3,7-dirhamnoside), which has significant therapeutic properties in the context of liver cancer treatment, is an active ingredient that attracts the attention of researchers due to its potential. The following study was aimed at identifying and analyzing potential targets and mechanisms of action of kaempferitrin in liver cancer using network pharmacology and molecular docking approaches, as well as confirming these targets and pathways in a mouse model with xenotransplanted tumors derived from SMMC-7721 cells. As a result of the analysis, 228 potential targets of kaempferitrin and 2186 specific targets for liver cancer were identified, of which 50 were common. A topological analysis of the network allowed us to identify eight key targets, among which SIRT1 and TP53 demonstrated high affinity for kaempferitrin, which was confirmed by molecular docking and molecular dynamics modeling. Cluster analysis of MCODE revealed the most significant functional module of the PPI network, including SIRT1 and TP53, which is mainly associated with cell apoptosis. Enrichment analysis by Gene Ontology (GO) and Kyoto Encyclopedia of Genes and Genomes (KEGG) has shown that kaempferitrin is capable of having a therapeutic effect on liver cancer, probably through stimulation of apoptosis through the p21/Bcl-2/caspase 3 signaling pathway. These hypotheses have been confirmed by experimental studies both in vitro and in vivo. The present study has not only expanded the understanding of the mechanisms of action of kaempferitrin in the context of liver cancer therapy, identifying key targets and signaling pathways, but also provided experimental evidence confirming the possibility of its clinical use in the treatment of this disease. The findings open up new perspectives for the development of innovative therapeutic strategies based on the use of kaempferitrin and its derivatives [42].

Resveratrol, a natural polyphenol found in grapes, nuts, wine and berries, is a promising agent for the prevention and treatment of cancer. Studies demonstrate its ability to reduce the negative effects of chemotherapy, as well as modulate key mechanisms of cell death, such as apoptosis and autophagy. These processes play a central role in the elimination of transformed cells, and resveratrol has a significant effect on their regulation by affecting a variety of signaling pathways and modulating the expression of the corresponding genes [43].

Thus, even a brief overview of the identified polyphenolic compounds in extracts of the species *P. nipponica* var. *kurilensis*, *P. sargentii*, and *P. maximowiczii* and the confirmation of their pharmacological properties allows us to conclude that these extracts have significant potential in the field of nutrition and their prospects as new therapeutic agents.

## 4. Materials and Methods

### 4.1. Plant Material

Nine *P. nipponica* var. *kurilensis*, *P. sargentii*, and *P. maximowiczii* varieties were included as plant material. The flowers from *P. nipponica* var. *kurilensis*, *P. sargentii*, and *P. maximowiczii* were collected in Komarov Botanical Institute’s Botanical Garden of Peter the Great, Saint Petersburg, Russia (N 59°58′12″, E 30°19′38″) (Table 3).

Three flowers were collected at the stage of full flowering of the plant. The samples were washed with distilled water and stored at −80 °C until processed. All samples morphologically corresponded to the pharmacopoeial standards of the State Pharmacopoeia of the Russian Federation.

### 4.2. Chemicals and Reagents

All chemicals used in this study were of analytical grade. High-performance liquid chromatography (HPLC)-grade acetonitrile was purchased from Fisher Scientific (Southborough, UK). Mass-spectrometry (MS)-grade formic acid was purchased from Sigma-Aldrich (Steinheim, Germany). Ultra-pure water was prepared by using a SIEMENS ULTRA clear (SIEMENS Water Technologies, Munich, Germany).

### 4.3. Fractional Maceration

The fractional maceration technique was used to obtain highly concentrated extracts [44]. Aqueous ethanol (80%) was used for extraction. From 150 g of the flowers, 20 g of flowers of each variety were randomly selected for maceration. The total amount of the extractant (aqueous ethanol 95%) was divided into 3 parts, and the parts of the plant were consistently infused with the first, second, and third parts. The infusion of each part of the extractant lasted seven days at room temperature. Three replicates of the extraction process were carried out on each plant sample. The extract was filtered through Whatman filter paper. The filtrates were diluted with acetonitrile to a final working concentration for analysis.

### 4.4. Liquid Chromatography

High Performance Liquid Chromatography was performed using a Shimadzu LC-20 Prominence HPLC (Shimadzu, Kyoto, Japan) equipped with a UV sensor and a C18 silica reverse phase column (4.6 × 150 mm, particle size: 2.7 μm) to perform the separation of multicomponent mixtures. The gradient elution program with two mobile phases (A, deionized water; B, acetonitrile with formic acid 0.1% *v*/*v*) was as follows: 0–2 min, 0% B; 2–50 min, 0–100% B; control washing 50–60 min, 100% B. The entire HPLC analysis was performed with a UV–vis detector SPD-20A (Shimadzu, Kyoto, Japan) at a wavelength of 230 nm for identification of compounds; the temperature was 25 °C, and the total flow rate was 0.25 mL min^−1^. The injection volume was 10 µL. Additionally, liquid chromatography was combined with a mass spectrometric ion trap to identify compounds.

### 4.5. Mass Spectrometry

Mass spectrometry analysis was performed on an ion trap amaZon SL (BRUKER DALTONIKS, Munich, Germany) equipped with an ESI source in positive and negative ion modes. The optimized parameters were obtained as follows: ionization source temperature: 70 °C, gas flow: 4 L/min, nebulizer gas (atomizer): 7.3 psi, capillary voltage: 4500 V, end plate bend voltage: 1500 V, fragmentary: 280 V, collision energy: 60 eV. An ion trap was used in the scan range *m*/*z* 100–1.700 for MS and MS/MS. The chemical constituents were identified by comparing their retention index, mass spectra, and MS fragmentation with an in-house self-built database (Biotechnology, Bioengineering and Food Systems Laboratory, Far-Eastern Federal University, Russia). The in-house self-built database is based on data from other spectroscopic techniques, such as nuclear magnetic resonance, ultraviolet spectroscopy, and MS, as well as data from the literature that is continuously updated and revised. The capture rate was one spectrum/s for MS and two spectrum/s for MS/MS. Data acquisition was controlled by Windows software for BRUKER DALTONIKS. All experiments were repeated three times. A four-stage ion separation mode (MS/MS mode) was implemented. This work is supported by the Programm “Priority-2030” of Tomsk State University.

### 4.6. Data Processing and Statistical Analysis

Raw peak area data of the successful runs were log-transformed to normalize the distribution. Subsequently, a presence/absence matrix was generated. All statistical analyses were performed in R version 4.5.3 (https://www.r-project.org/) with the following packages: readxl for importing Excel data files, ggplot for creating publication-quality graphics, pheatmap for generating heatmaps with hierarchical clustering, dplyr and tidyr for data manipulation and reshaping, and writexl for exporting results. R version 4.5.3 with the packages readxl, ggplot2, pheatmap, dplyr, and tidyr was used for all statistical analyses. To see sample clustering patterns, principal component analysis (PCA) was performed on the log-transformed data with mean-centering and scaling. Euclidean distance and Ward’s technique were used to accomplish hierarchical clustering, which was displayed as a heatmap with row scaling. Bar plots and heatmaps were used to display the number of compounds by chemical class for each species and geographic origin in the presence/absence data.

## 5. Conclusions

Based on the results of high-performance liquid chromatography with electrospray ionization (HPLC-ESI-MS) and tandem mass spectrometry (ESI-MS/MS), we concluded that extracts of *P. nipponica* var. *kurilensis*, *P. sargentii*, and *P. maximowiczii* are characterized by a high content of polyphenols. One hundred and twenty-two compounds were identified during the analysis, of which one hundred and eight are polyphenols. The identified polyphenols are dominated by flavones, flavanols, flavan-3-ols, and anthocyanins. In addition, the extracts contain compounds belonging to twenty different chemical classes, including indole-sesquiterpene alkaloids, iridoid glucosides, phenylpropanoid glucosides, amino acids and their derivatives, nucleotides and omega-hydroxyamino acids. It should be noted that the data on the metabolome of secondary metabolites of *P. nipponica* var. *kurilensis*, *P. sargentii*, and *P. maximowiczii* were obtained for the first time. The richness and complexity of the polyphenolic composition of these extracts indicate their significant potential for use in the pharmaceutical industry, as well as for the development of innovative drug strategies.

## Figures and Tables

**Table 1:** The Jaccard index, which is a metric for assessing similarities and differences in the composition of polyphenolic compounds, shows the comparative analysis of three species of the genus Prunus (*P. nipponica* var. *kurilensis*, *P. sargentii*, *P. maximowiczii*).

	*P. sargentii* (71)	*P. nipponica var kurilensis* (58)	*P. maximowiczii* (55)
***P. sargentii*** (71)		48 0.5393	32 0.3404
***P. nipponica* var. *kurilensis*** (58)	48 0.5393		26 0.2626
***P. maximowiczii*** (55)	32 0.3404	26 0.2626	

**Table 2:** The distribution of the polyphenol compounds in *P. nipponica* var. *kurilensis*, *P. sargentii*, and *P. maximowiczii*.

Varieties	Similarities	Polyphenol Group
*P. maximowiczii*; *P. nipponica* var. *kurilensis*; *P. sargentii*	21	Rutin; Umbelliferone; Umbelliferone hexoside; Isorhamnetin; Delphinidin 3-O-hexoside; Ellagic acid; Kaempferol 3-O-deoxyhexoside-hexoside; Caffeic acid; Quercetin 3-glucoside; Methylgallic acid; Ethyl protocatechuate; Delphinidin 3-O-glucoside; Quercetin-O-rhamnoside-O-hexoside; Delphinidin 3-O-(6-O-p-coumaroyl) glucoside; Quercetin-7-O-Beta-Glucopyranoside; Delphinidin 3-O-rutinoside; Isorhamnetin-di-O-hexoside; Quercetin-3-O-hexoside; Delphinidin 3-O-beta-galactoside; Caffeic acid-O-hexoside; Kaempferol 3-O-rutinoside
*P. nipponica* var. *kurilensis*; *P. sargentii*	21	Rhamnetin-di-O-hexoside; Herbacetin; Catechin; (epi)-Afzelechin; 3,4-Dihydroxyhydrocinnamic acid; Genkwanin; (Epi)-afzelechin derivative; Afzelechin; (Epi)-catechin derivative 3; (Epi)-catechin; (Epi)-catechin derivative; Protocatechuic acid; (Epi)-catechin derivative 2; Ellagic acid-O-hexoside; Caftaric acid; Ethyl caffeate; Apigenin-7-O-glucoside; Caffeic acid hexoside dimer; Myricetin; Kaempferol rhamnosyl glucoside; Apigenin 4′-O-hexoside;
*P. maximowiczii*; *P. sargentii*	12	Kaempferol; Ferulic acid-O-hexoside; p-Coumaric acid-O-hexoside; Resveratrol; Quercetin; Ferulic acid; Petunidin 3,5-dihexoside; Syringaresinol; Bioquercetin; Cyanidin-3-O-rutinoside; Petunidin 3-O-glucoside-5-O-glucoside; Fraxetin
*P. maximowiczii*; *P. nipponica* var. *kurilensis*	2	Kaempferol-3-O-hexoside; Astragalin
*P. sargentii*	17	Kaempferol-3-O-coumaroylhexoside; Mosloflavone; Caffeoylmalic acid; Kaempferol-deoxy-hexoside; Malvidin 3-O-(6″-O-p-coumaroyl)glucoside; Quercetin O-hexoside O-deoxyhexoside; p-Coumaric acid; Vanillic acid-O-hexoside; Apigenin-7-O-hexoside; Quercetin 7-O-rutinoside; Myricitrin; Tricin O-rhamnoside; Jaceosidin; Caffeic acid derivative; Kaempferol 3-O-robinobioside; Malvidin 3-O-(6″-O-p-coumaroyl)hexoside; Formononetin
*P. nipponica* var. *kurilensis*	14	Isorhamnetin 3-O-(6″-O-rhamnosyl-hexoside); Acacetin O-glucoside; Luteolin-O-hexoside; Petunidin 3-O-p-coymaroyl hexoside; Cirsiliol; Sakuranetin; Methyl ellagic acid valerylpentose; Acacetin 7-O-glucoside; Petunidin 3-O-(6″-caffeoyl)hexoside; Dihydroxy-dimethoxy(iso)flavone; Luteolin 7-O-glucoside; Isorhamnetin-3-O-rutinoside; Calycosin; Isorhamnetin O-rhamnosyl-hexoside
*P. maximowiczii*	20	Kaempferol 3,7-dirhamnoside; Delphinidin-3,5-diglucoside; Delphinidin-3-caffeoylglucoside; Quercetin-O-dihexoside; Vimalin; Cyanidin O-deoxyhexoside; Ellagic acid-rhamnoside; Matairesinol; Kaempferol 3-O-pentoside; Quercetin O-(caffeyl)hexoside; Kaempferol-3,7-O-diglucoside; Quercetin 3-O-pentoside; Afzelin; Avicularin; Quercetin-3-O-arabinoside; Chlorogenic acid; Ellagic acid-O-deoxyhexoside; Quercetin-3-alpha-xylopyranoside; Kaempferol 3-O-glucosyl-7-O-rhamnoside; Scopoletin

**Table 3:** Origin of test varieties.

No.	Variety	Origin and Year of Receipt
1	*Prunus sargentii*	European nurseries, 2014
3	*Prunus sargentii*	European nurseries, 2014
5	*Prunus sargentii*	Hokkaido Island (Japan), 2003
9	*Prunus sargentii*	Hokkaido Island (Japan), 2003
10	*Prunus sargentii*	Hokkaido Island (Japan), 2003
15	*Prunus sargentii*	Kuril Islands (Russia), 1982
7	*Prunus nipponica* var. *kurilensis*	Kuril Islands (Russia), 1982
31	*Prunus maximowiczii*	Sakhalin Island (Russia),1977

## Data Availability

The original contributions presented in this study are included in the article. Further inquiries can be directed to the corresponding authors.

## Appendix A

Approximate comparison of chemical constituents identified in extracts of *P. nipponica* var. *kurilensis*, *P. sargentii*, and *P. maximowiczii* flowers.

**Table A1:** Chemical compounds identified from the extracts of *P. nipponica* var. *kurilensis*, *P. sargentii*, and *P. maximowiczii,* in positive and negative ionization modes by HPLC-ion trap-MS/MS.

	Class of Compounds	Identification	Formula	Calculated Mass	Observed Mass [M − H]^−^	Observed Mass [M + H]^+^	MS/MS Stage 2 Fragmentation	MS/MS Stage 3 Fragmentation	MS/MS Stage 4 Fragmentation	Scientific Sources with Mass Spectrometric Data
1	Flavone	**Formononetin** [Biochanin B; Formononetol]	C_16_H_12_O_4_	268.26		269.11	209.06; 195.07; 169.16; 127.02	173.16; 133.11		*Dracocephalum jacutense* [45]
2	Flavone	**Genkwanin** [Gengkwanin; Puddumetin; Apigenin 7-Methyl Ether]	C_16_H_12_O_5_	284.26		285.12	267.16; 229.06; 167.04			*Rosmarinus officinalis* [25]
3	Flavone	**Calycosin** [3′-Hydroxyformononetin]	C_16_H_12_O_5_	284.26		285.09	224.08; 167.07			Huolisu Oral Liquid [46]; *Astragali Radix* [23]
4	Flavone	**Mosloflavone**	C_17_H_14_O_5_	298.29		299.15	238.96	173.08		*Actinocarya tibetica* [47]
5	Pentahydroxyflavone	**Herbacetin** [3,5,7,8-Tetrahydroxy-2-(4-hydro- xyphenyl)-4H-chromen-4-one]	C_15_H_10_O_7_	302.23		303.08	203.06; 185.05; 140.05	156.99		*Triticum aestivum* [48]; *Loropetalum chinense* [34]
6	Flavone	**Dihydroxy-dimethoxy(iso)flavone**	C_17_H_14_O_6_	314.28		315.31	297.25; 268.17; 218.11	224.15; 202.21		propolis [26]; *Astragali Radix* [23]; *Rosmarinus officinalis* [25]
7	Flavone	**Cirsiliol**	C_17_H_14_O_7_	330.28		331.06	316.03	298.05; 268.95; 215.86	270.06	*Juglans mandshurica* [49]; *Rosa acicularis*, *Rosa rugosa*; *Rosa amblyotis* [20]; *Inula viscosa* [21]
8	Flavone	**Jaceosidin** [5,7,4′-trihydroxy-6′,5′-dimethoxyflavone]	C_17_H_14_O_7_	330.28		331.05	303.07; 285.03; 257.01; 185.02			*Artemisia argyi* [50]; *Rosa acicularis*, *Rosa rugosa*; *Rosa amblyotis* [20]
9	Flavone	**Apigenin 4′-O-hexoside**	C_21_H_20_O_10_	432.37	431		269	225	181	*Strawberry* [51]; *Lonicera henryi* [12]
10	Flavone	**Apigenin-7-*O*-glucoside** [Apigetrin; Cosmosiin]	C_21_H_20_O_10_	432.37	431.15		269.07; 257.16;	227.11; 157.17	215.09	*Lonicera henryi* [12]; *Loropetalum chinense* [34]
11	Flavone	**Apigenin-7-*O*-hexoside**	C_21_H_20_O_10_	432.37	431.15		269.07; 257.16;	227.11; 157.17	215.09	*Bituminaria* [52]; *Stevia rebaudiana* [24]
12	Flavone	**Luteolin 7-*O*-glucoside** [Cynaroside]	C_21_H_20_O_11_	448.37		449.01	287.06	213.03		*Lonicera japonica* [13]; *Lonicera henryi* [12]
13	Flavone	**Luteolin-*O*-hexoside** [Tetrahydroxyflavone-*O*-hexoside]	C_21_H_20_O_11_	448.37		449	287	213; 137	213; 185; 157	*Bituminaria* [52]; *Rosa rugosa*; *Rosa amblyotis* [20]; *Ribes meyeri* [11]
14	Flavone	**Acacetin O-glucoside**	C_22_H_22_O_10_	446.40		447.06	285.08	257.10; 167.05		Mexican lupine species [16]
15	Flavone	**Acacetin 7-O-glucoside** [Tilianin]	C_22_H_22_O_10_	446.40		447.06	285.08	257.11; 229.12; 167.09	185.99	Bougainvillea [53]
16	Flavonol	**Kaempferol**	C_15_H_10_O_6_	286.23		287.07	240.99; 187.09	205.05; 169.11		*Rosa rugosa*; *Rosa amblyotis* [20]; *Ribes meyeri* [11]; *Lonicera japonica* [13]; Andean blueberry [14]; *Juglans mandshurica* [49];
17	Flavonol	**Quercetin**	C_15_H_10_O_7_	302.23		303.03	257.09; 165.04	228.71; 157.05	201.10	propolis [26]; *Ribes meyeri* [11]
18	Flavonol	**Isorhamnetin** [Isorhamnetol; Quercetin 3′-Methyl ether; 3-Methylquercetin]	C_16_H_12_O_7_	316.26		318	302.02	285.03; 228.07	256.99	*Rosa acicularis*; *Rosa rugosa*; *Rosa amblyotis* [20]; *Phoenix dactylifera* [27]; Andean blueberry [14]; *Inula viscosa* [21]
19	Flavonol	**Myricetin**	C_15_H_10_O_8_	318.23		319.05	273.08; 219.06	191.05; 173.05; 143.05	161.10; 143.06; 125.10	*Juglans mandshurica* [49]; Andean blueberry [14]
20	Flavonol	**Kaempferol 3-O-pentoside**	C_20_H_18_O_10_	418.35		419.30	287.21	258.16; 133.13		*Stevia rebaudiana* [24]; *Rosa rugosa*; *Rosa amblyotis* [20]; Andean blueberry [14]
21	Flavonol	**Afzelin** [Kaempferol-3-*O*-Rhamnoside; Kaempferin]	C_21_H_20_O_10_	432.37		433.26	287.18	165.01		*Juglans mandshurica* [49]; *Loropetalum chinense* [34]
22	Flavonol	**Kaempferol-deoxy-hexoside**	C_21_H_20_O_10_	432.37	431.18		257.15	227.14; 157.12	215.14	*Hibiscus rosa sinensis* [54]; *Grape* [31]
23	Flavonol	**Avicularin** (Quercetin 3-*Alpha*-L-Arabinofuranoside; Avicularoside)	C_20_H_18_O_11_	434.35	433.34		301.21	271.21; 179.13	229.18	*Juglans mandshurica* [49]; *Ribes meyeri* [11]; *Rosa rugosa*; *Rosa amblyotis* [20]; propolis [26]; *Loropetalum chinense* [34]
24	Flavonol	**Quercetin-3-*alpha*-xylopyranoside** [Quercetin 3-*O*-xylopyranoside]	C_20_H_18_O_11_	434.35		435.27	303.19	257.16; 165.10	229.18	*Vaccinium myrtillus* [55]; *Rosa acicularis*; *Rosa amblyotis* [14]
25	Flavonol	**Quercetin 3-*O*-pentoside**	C_20_H_18_O_11_	434.35	433.34		301.21	271.21; 179.13	229.18	*Cuphea ignea* [56]; *Ribes meyeri* [11]; *Rosa rugosa*; *Rosa amblyotis* [20]
26	Flavonol	**Quercetin-3-O-arabinoside** [Guaijaverin; Guajaverin]	C_20_H_18_O_11_	434.35	433.34		301.21	271.21; 179.13	229.18	*Vaccinium myrtillus* [55]; *Lonicera henryi* [12]; Embelia [18]; *Rosa rugosa*; *Rosa amblyotis* [20]
27	Flavonol	**Astragalin** [Kaempferol 3-O-glucoside; Astragaline]	C_21_H_20_O_11_	448.38		449.01	287.06	241.06; 165.00	213.03	*Juglans mandshurica* [49]; *Ribes meyeri* [11]; *Lonicera japonica* [13]
28	Flavonol	**Kaempferol-3-O-hexoside**	C_21_H_20_O_11_	448.38		449.02	287	287; 231	230; 111	*Cuphea ignea* [56]; Andean blueberry [14]; *Rhus coriaria* [15]
29	Flavonol	**Quercetin 3-glucoside** [Isotrifoliin; Isoquercetin; Isoquercitrin; Hirsutrin]	C_21_H_20_O_12_	464.38		465.46	303.05	257.02; 265.07	228.98	*Rubus occidentalis* [10]; *Ribes meyeri* [11]; *Lonicera henryi* [12]; *Lonicera japonica* [13]; Andean blueberry [14]; *Rosa rugosa*; *Rosa amblyotis* [20]
30	Flavonol	**Quercetin 3-hexoside**	C_21_H_20_O_12_	464.38		465.31	303.25	257.23; 265.17	229.02	Andean blueberry [14]; *Rosa rugosa*; *Rosa amblyotis* [20]
31	Flavonol	**Quercetin-7-O-Beta-Glucopyranoside**	C_21_H_20_O_12_	464.38		465.2	303.45	257.34; 265.22	229.17	*Rosa acicularis; Rosa rugosa*; *Rosa amblyotis* [20]
32	Flavonol	**Myricitrin** [Myricitroside; Myricetin 3-O-Rhamnoside]	C_21_H_20_O_12_	464.38		465	318.82	217.03	171.04	*Juglans mandshurica* [49]; *Loropetalum chinense* [34]; *Rhus coriaria* [15]
33	Flavonol	**Tricin *O-*rhamnoside**	C_22_H_20_O_12_	476.39		477.05	331.08	285.04; 184.51	203.02	*Loropetalum chinense* [34]
34	Flavonol	**Kaempferol 3,7-dirhamnoside** [Kaempferitrin]	C_27_H_30_O_14_	578.52		579.00	433.28	287.18	165.02	*Camellia kucha* [57]; Huolisu Oral Liquid [46]; *Loropetalum chinense* [34]
35	Flavonol	**Kaempferol-3-*O*-coumaroylhexoside**	C_27_H_30_O_15_	594.52		595	287.05	241.06; 165.01		*Ribes dikuscha* [58]; *Rosa rugosa*; *Rosa amblyotis* [20]
36	Flavonol	**Kaempferol 3-O-rutinoside [Nicotiflorin]**	C_27_H_30_O_15_	594.52		595.38	287.17; 449.28	241.20; 165.06		*Triticum aestivum* [48]; *Ribes meyeri* [11]; *Lonicera japonica* [13]; *Rhus coriaria* [15]; *Loropetalum chinense* [34]
37	Flavonol	**Kaempferol rhamnosyl glucoside**	C_27_H_30_O_15_	594.52		595	287.03; 448.93	240.99; 165.06	213.07	*Tilla tomentosa* [59]; *Rosa rugosa*; *Rosa amblyotis* [20]
38	Flavonol	**Kaempferol 3-*O*-deoxyhexoside-hexoside**	C_27_H_30_O_15_	594.52		595.28	433.31; 287.18	241.09; 165.10		*Hibiscus rosa sinensis* [54]; *Rosa rugosa*; *Rosa amblyotis* [20]; *Stevia rebaudiana* [24]
39	Flavonol	**Kaempferol 3-O-glucosyl-7-O-rhamnoside**	C_27_H_30_O_15_	594.52		595.15	433.31; 287.18	241.09; 165.10	136.94	*Pear* [60]
40	Flavonol	**Kaempferol 3-O-robinobioside**	C_27_H_30_O_15_	594.52		595.21	433.2; 287.1	241.15	137	*Loropetalum chinense* [34]
41	Flavonol	**Rutin** (Quercetin 3-*O*-rutinoside)	C_27_H_30_O_16_	610.52		611.39	303.20; 465.23	257.11; 165.04		*Ribes magellanicum* [61]; *Rubus occidentalis* [10]; *Ribes meyeri* [11]; *Lonicera henryi* [12]; *Lonicera japonica* [13]
42	Flavonol	**Quercetin 7-O-rutinoside**	C_27_H_30_O_16_	610.52		611	303.03; 465.03	257.03; 165.05	229.06	*Triticum aestivum* [48]; *Rosa rugosa*; *Rosa amblyotis* [20]
43	Flavonol	**Quercetin-O-rhamnoside-O-hexoside**	C_27_H_30_O_16_	610.52		611.39	303.06; 465.01	257.11; 229.14; 165.04	229.09; 161.11	*Papaya*; *Strawberry* [51]; *Ribes dikuscha* [58]; *Rosa rugosa*; *Rosa amblyotis* [20]
44	Flavonol	**Quercetin O-hexoside O-deoxyhexoside**	C_27_H_30_O_16_	610.52		611	303.03;	257.04; 165.04	229.05	*Ribes dikuscha* [58]; *Rosa rugosa*; *Rosa amblyotis* [20]; Andean blueberry [14]
45	Flavonol	**Bioquercetin** [Quercetin-3-*O*-robinobioside]	C_27_H_30_O_16_	610.52		610.90	303.04; 464.98	257.00; 165.01	229.02; 201.05	*Ribes dikuscha* [58]; *Rosa rugosa*; *Rosa amblyotis* [20]; *Aspalathus linearis* [19]
46	Flavonol	**Kaempferol-3,7-O-diglucoside**	C_27_H_30_O_16_	610.52		610.21	449.29; 287.20	287.20	241.12; 165.08	Rosemary; Mint; Salvia; Thymus vulgaris; Laurus; nobilis [30]; Rapeseed petals [28]; *Taraxacum officinale* [29]
47	Flavonol	**Isorhamnetin-3-O-rutinoside** [Narcissin; Narcissoside]	C_28_H_32_O_16_	624.54		625	317	302; 139	273	Lemon; *Papaya*; *Strawberry* [51]; *Ribes aureum*, *Ribes triste*, *Ribes dikuscha* [58]; Embelia [18]
48	Flavonol	**Isorhamnetin 3-O-(6** **″-*O*-rhamnosyl-hexoside)**	C_28_H_32_O_16_	624.54		625	317	302		*Lonicera henryi* [12]; *Vitis vinifera* [36]
49	Flavonol	**Isorhamnetin O-rhamnosyl-hexoside**	C_31_H_28_O_14_	624.54		625	317	302; 139	273	propolis [26]
50	Flavonol	**Quercetin-** ***O*** **-dihexoside**	C_27_H_30_O_17_	626.52		627.37	303.22; 465.30	257.15; 165.09	229.17; 201.14	*Phoenix dactylifera* [27]; Andean blueberry [14]; *Rosa rugosa*; *Rosa amblyotis* [20]; *Inula viscosa* [21]
51	Flavonol	**Quercetin O-(caffeyl)hexoside**	C_30_H_26_O_15_	626.52		627.37	303.22; 465.30	257.15; 165.09	229.17; 201.14	*Inula viscosa* [62]
52	Flavonol	**Rhamnetin-di-*O*-hexoside**	C_28_H_32_O_17_	640.54	639.15		315.11	299.07	271.07	*Ribes fragrans* [63]
53	Flavonol	**Isorhamnetin-di-O-hexoside** [Methyl quercetin-O-dihexoside]	C_28_H_32_O_17_	640.54		641	317.05	302.03	283.98; 228.02; 168.98	Passion fruit; *Papaya*; *Strawberry* [51]; *Phoenix dactylifera* [27]
54	Flavanone	**Sakuranetin** [7-O-Methylnaringenin; 5,4′-Dihydroxy-7-Methoxyflavanone]	C_16_H_14_O_5_	286.28		287.07	259.16; 219.55; 169.09			Jatropha [64]; *Loropetalum chinense* [34]
55	Flavan-3-ol	**Afzelechin**	C_15_H_14_O_5_	274.27		275.14	245.12; 175.16	175.04; 157.08; 127.03	126.92	*Camellia kucha* [57]; *Rosa rugosa*; *Rosa amblyotis* [20]; *Loropetalum chinense* [34]
56	Flavan-3-ol	**(epi)-Afzelechin**	C_15_H_14_O_5_	274.27		275.09	245.09; 175.09	157.03; 123.09		*A. cordifolia*; *F. glaucescens*; *F. herrerae* [65]; *Loropetalum chinense* [34]; *Rosa rugosa*; *Rosa amblyotis* [20]
57	Flavan-3-ol	**Catechin**	C_15_H_14_O_6_	290.27		291.14	261.11; 157.13	173.06	143.05	*Ribes magellanicum* [61]; *Ribes meyeri* [11]
58	Flavan-3-ol	**(Epi)-catechin**	C_15_H_14_O_6_	290.27		291.10	272.97; 261.05; 243.05	173.05; 160.99; 125.10	143.09; 125.11	*Vaccinium myrtillus* [55]; *Rubus occidentalis* [10]; Andean blueberry [14]
59	Flavan-3-ol	**(Epi)-catechin derivative**		378.37		379.12	291.1	261.19; 173.22	144	*PubChem*
60	Flavan-3-ol	**(Epi)-afzelechin derivative**	C_18_H_16_O_10_	392.31		393.12	275.08; 245.10; 149.02	245.07; 175.08	175.07; 157.04	*Zostera marina* [66]
61	Flavan-3-ol	**(Epi)-catechin derivative 2**	C_18_H_16_O_11_	408.31		409.13	291.11	261.09; 173.04	143.06	*PubChem*
62	Flavan-3-ol	**(Epi)-catechin derivative 3**		424.23		425.14	291.09	261.11; 173.07	143.03	*PubChem*
63	Phenylpropanoid (cinnamic alcohol glycoside)	**Vimalin**	C_16_H_22_O_7_	326.34		327.37	309.34; 251.22; 217.27	208.18		*Rosa rugosa*; *Rosa amblyotis* [20]
64	Iridoid glucoside	**Harpagide**	C_15_H_24_O_10_	364.34		365.01	203			*Honey* [67]
65	Anthocyanin	**Cyanidin O-deoxyhexoside**	C_21_H_21_O_10_	433.38		433.28	287.19	241.19; 213.18; 165.05	213.09; 200.05	Andean blueberry [14]
66	Anthocyanin	**Delphinidin 3-O-glucoside** [Mirtillin]	C_21_H_21_O_12+_	465.39		465	303.05	285; 01; 257.02; 165.07	228.98	*Ribes dikuscha* [58]; *Rosa acicularis*; *Rosa rugosa*; *Rosa amblyotis* [20]; *Ribes magellanicum* [61]
67	Anthocyanin	**Delphinidin 3-O-hexoside**	C_21_H_21_O_12+_	465.39	463		301	255; 229; 179; 151	185; 147	*Ribes dikuscha* [58]; *Rosa acicularis*; *Rosa rugosa*; *Rosa amblyotis* [20]; Andean blueberry [14]
68	Anthocyanin	**Delphinidin 3-*O-beta-*galactoside**	C_21_H_21_O_12+_	465.39		465.28	303.20	257.20; 165.11	229.12; 201.10	*Ribes dikuscha* [58]; *Rosa acicularis*; *Rosa rugosa*; *Rosa amblyotis* [20]
69	Anthocyanin	**Cyanidin-3-O-rutinoside** [Keracyanin; Antirrhinin; Sambucin]	C_27_H_31_O_15_	595.53		595	287.03; 448.93	240.99; 165.06	213.07	*Rosa rugosa*; *Rosa amblyotis* [20]; *Ribes magellanicum* [61]
70	Anthocyanin	**Delphinidin 3-O-rutinoside** [Tulipanin; Delphinidin 3-Rhamnosyl-Glucoside]	C_27_H_31_O_16_	611.53		611.39	465.23; 303.20	257.11; 229.14; 165.04	161.11	*Berberis ilicifolia*; *Berberis empetrifolia*; *Ribes magellanicum*; *Ribes cucullatum Myrteola nummalaria* [68]; *Ribes aureum*; *Ribes triste* [50]; *Rosa rugosa*; *Rosa amblyotis* [20]
71	Anthocyanin	**Delphinidin 3-*O*-(6-*O-p*-coumaroyl) glucoside**	C_30_H_27_O_14_	611.53		611	465.00; 303.03	257.04; 165.04	229.05	*Ribes aureum*; *Ribes triste* [50]; *Rosa rugosa*; *Rosa amblyotis* [20]; *Grape* [31]; *Vitis vinifera* [32]
72	Anthocyanin	**Petunidin 3-*O*-(*6*″-caffeoyl)hexoside**	C_31_H_29_O_14_	625.55		625	317	302; 139	273	*Vitis vinifera* [36]
73	Anthocyanin	**Petunidin 3-O-p-coymaroyl hexoside**	C_28_H_33_O_16_	625.55		625	317	302; 139	273	*Ribes dikuscha* [58]; Grape vine varieties [35]
74	Anthocyanin	**Delphinidin-3-caffeoylglucoside**	C_30_H_27_O_15_	627.53		627.37	303.22; 465.30	257.15; 165.09	229.17; 201.14	*Triticum aestivum* [69]; *Rosa rugosa*; *Rosa amblyotis* [20]
75	Anthocyanin	**Delphinidin-3,5-diglucoside**	C_27_H_31_O_17_	627.53		627.37	303.22; 465.30	257.15; 165.09	229.17; 201.14	*Berberis microphylla* [70]; *Rosa rugosa*; *Rosa amblyotis* [20]
76	Anthocyanin	**Malvidin 3-O-(6″-O-p-coumaroyl)glucoside**	C_32_H_31_O_14_	639.58		639.90	317.06	302.04	283.93; 228.10	*Grape* [31]; *Vitis vinifera* [32]
77	Anthocyanin	**Malvidin 3-*O*-(6″-*O*-*p*-coumaroyl)hexoside**	C_32_H_31_O_14_	639.58		639.90	317.06	302.04	283.93; 228.10	Grape vine varieties [35]; *Vitis vinifera* [36]
78	Anthocyanin	**Petunidin 3-O-glucoside-5-O-glucoside**	C_28_H_33_O_17_	641.55		641	317.05	302.03	283.98; 228.02; 168.98	*Loropetalum chinense* [34]; *Grape* [31]; *Vitis vinifera* [32]
79	Anthocyanin	**Petunidin 3,5-dihexoside**	C_28_H_33_O_17_	641.55		641	317.05	302.03	283.98; 228.02; 168.98	*Berberis microphylla* [70]
80	Hydroxybenzoic acid (Phenolic acid)	**Protocatechuic acid**	C_7_H_6_O_4_	154.12		155.08	127.09			*Camellia kucha* [57]; *Rosa rugosa*; *Rosa amblyotis* [20]; *Ribes meyeri* [11]; *Lonicera japonica* [13]; *Rhus coriaria* [15]
81	Hydroxycinnamic acid	**p-Coumaric acid** [4-Hydroxycinnamic acid]	C_9_H_8_O_3_	164.16		165.10	145.11	127.14		*Juglans mandshurica* [49]; *Rubus occidentalis* [10]; *Ribes meyeri* [11]; Andean blueberry [14]
82	Hydroxycinnamic acid	**Caffeic acid** [(2E)-3-(3,4-Dihydroxyphenyl)acrylic acid]	C_9_H_8_O_4_	180.16		181.07	163.06; 145.08			*Juglans mandshurica* [49]; *Rubus occidentalis* [10]; *Ribes meyeri* [11]; *Lonicera japonica* [13]
83	Hydroxycinnamic acid	**3,4-Dihydroxyhydrocinnamic acid** [Dihydrocaffeic acid]	C_9_H_10_O_4_	182.17		183.11	165.09; 147.10; 129.14	129.08; 117.08		*Eucalyptus globulus* [71]
84	Dihydrobenzoic acid	**Ethyl protocatechuate** [3,4-Dihydroxybenzoic Acid Ethyl Ester]	C_9_H_10_O_4_	182.17		183.10	155.06	127.07		*Ocimum* [72]; *Rosa rugosa*; *Rosa amblyotis* [20]
85	Phenolic acid	**Methylgallic acid** [Methyl gallate; Methyl 3,4,5-trihydroxybenzoate]	C_8_H_8_O_5_	184.15		185.11	156.07; 143.11; 127.09	127.12		*Papaya*; *Strawberry* [51]; *Rosa rugosa*; *Rosa amblyotis* [20]; Andean blueberry [14]; *Rhus coriaria* [15]
86	Trans-cinnamic acid	**Ferulic acid**	C_10_H_10_O_4_	194.18		195.02	177.10	145.07		*Juglans mandshurica* [49]; *Lonicera japonica* [13]; Andean blueberry [14]; *Astragali Radix* [23]
87	Hydroxycinnamic acid	**Ethyl caffeate** [Ethyl 3,4-Dihydroxycinnamate]	C_11_H_12_O_4_	208.21	207.13		179.08; 135.12	135.09		*Tilla tomentosa* [59]; *Ocimum* [72]
88	Hydroxycinnamic acid	**Caffeoylmalic acid**	C_13_H_12_O_8_	296.23	295.14		179.09	135.12		*Strawberry* [51]; *Inula viscosa* [62]
89	Hydroxybenzoic acid (Phenolic acid)	**Ellagic acid** [Benzoaric acid; Elagostasine; Lagistase; Eleagic acid]	C_14_H_6_O_8_	302.19		303.09	257.09;	245; 13; 175.12	175.10	*Juglans mandshurica* [49]; *Rosa rugosa*; *Rosa amblyotis* [20]; *Rubus occidentalis* [10]; *Ribes meyeri* [11]; *Rhus coriaria* [15]
90	Hydroxycinnamic acid	**Caftaric acid** [Cis-Caftaric acid; 2-Caffeoyl-L-Tartaric acid; Caffeoyl Tartaric acid]	C_13_H_12_O_9_	312.23		313.16	180.10; 145.07;	126.24		*G. linguiforme, F. herrerae* [65]: *Rhus coriaria* [15]
91	Hydroxycinnamic acid	**p-Coumaric acid-O-hexoside** [Trans-p-Coumaric acid 4-glucoside]	C_15_H_18_O_8_	326.29	325.16		163.14; 145.12			*Ribes dikuscha* [58]; *Ribes meyeri* [11]; Andean blueberry [14]; *Inula viscosa* [21]
92	Phenolic acid	**Vanillic acid-*O*-hexoside**	C_14_H_18_O_9_	330.29		331.06	185.03; 203.01; 257.02			*Punica granatum* [73]
93	Phenolic acid	**Caffeic acid-O-hexoside** [Caffeoyl-O-hexoside]	C_15_H_18_O_9_	342.29	341.15		179.07; 161.09; 135.09	135.13		Bougainvillea [53]; *Phoenix dactylifera* [27]; Andean blueberry [14]; Embelia [18]; *Inula viscosa* [21]
94	Hydroxycinnamic acid;	**Chlorogenic acid** [3-O-Caffeoylquinic acid]	C_16_H_18_O_9_	354.31		355.28	163.10	145.07	117.20	*Vaccinium myrtillus* [55]; *Lonicera henryi* [12]; *Lonicera japonica* [13]; Andean blueberry [14]
95	Phenolic acid	**Ferulic acid-*O*-hexoside**	C_16_H_20_O_9_	356.32	355.19		193.09; 217.10	134.13		*A. cordifolia*, *F. glaucescens*; *F. herrerae* [65]; Andean blueberry [14]
96	Phenolic acid	**Caffeic acid derivative**	C_18_H_18_O_9_	378.33	377.13		341.13	179.09; 161.10	143.09; 119.00	Bougainvillea [53]; Embelia [18]; *Rosa rugosa*; *Rosa amblyotis* [20]
97	Phenolic acid	**Ellagic acid-*O*-deoxyhexoside**	C_20_H_16_O_12_	448.33		449.28	303.18	257.14; 229.12; 165.15	229.13; 161.12	*Punica granatum* [73]; *Rosa rugosa*; *Rosa amblyotis* [20]
98	Phenolic acid	**Ellagic acid-rhamnoside**	C_20_H_16_O_12_	448.33		449.28	303.20	257.20; 165.11	229.14; 165.18	*Eucalyptus globulus* [71]
99	Phenolic acid	**Ellagic acid-*O*-hexoside**	C_20_H_16_O_13_	464.33		465.28	303.20	257.15; 165.07	229.10; 201.96	*Ribes dikuscha* [58]; *Rosa rugosa*; *Rosa amblyotis* [20]
100	Phenolic acid	**Methyl ellagic acid valerylpentose**	C_25_H_24_O_13_	532.45		533	303.03; 213.12	257.08; 165.06	229.06; 201.05	*Rubus occidentalis* [10]
101	Phenolic acid	**Caffeic acid hexoside dimer**		684.23	683.22		341.12	179.08	135.08	*Strawberry*, *Lemon*, *Cherimoya*, *Passion fruit*, *Strawberry* [51]
102	Stilbene	**Resveratrol** [trans-Resveratrol; 3,4′,5-Trihydroxystilbene; Stilbentriol]	C_14_H_12_O_3_	228.24		229	205.06; 184.03	166.04	124.19	*A. cordifolia*; *F. glaucescens*; *F. herrerae* [65]; *Ribes pauciflorum*, *Ribes dikuscha* [58]
103	Hydroxycoumarin	**Umbelliferone** [Skimmetin; Hydragin]	C_9_H_6_O_3_	162.14		163.08	145.05; 135.08	117.13		*F. glaucescens, F. herrerae* [65]; *Ribes aureum*; *Ribes pauciflorum*, *Ribes dikuscha* [58]
104	Coumarin	**Scopoletin** [Scopoletine; Gelseminic acid; Chrysatropic acid]	C_10_H_8_O_4_	192.17		193.19	147.07; 165.11	119.06		Cranberry [74]; Jatropha [64]
105	Coumarin	**Fraxetin**	C_10_H_8_O_5_	208.17		209.10	163.05	145.05		Jatropha [64]; *Ribes dikuscha* [58]; Embelia [18]
106	Coumarin	**Umbelliferone hexoside**	C_15_H_16_O_8_	324.28		325.11	288.37; 253.03; 223; 163.04	271.05; 253.10; 223.09	223.09; 195.06; 168.93	*G. linguiforme*, *F. herrerae* [65]; *Rosa rugosa*; *Rosa amblyotis* [20]
107	Lignans	**Matairesinol** [(-)-Matairesinol; Artigenin Congener]	C_20_H_22_O_6_	358.39		359.30	207.13; 123.09	165.07	123.11	Lignans [75]
108	Lignans	**Syringaresinol**	C_22_H_26_O_8_	418.44		419.07	326.04; 302.10; 273.04	298.02; 283.96; 254.12	180.03	Lignans [75]; *Rosa rugosa*; *Rosa amblyotis* [20]
	Others									
109	Amino acid	**L-theanine** [Theanine; Theanin; N-Ethyl-L-glutamine]	C_7_H_14_N_2_O_3_	174.19		157.11				*Camellia kucha* [57]; *Rosa rugosa*; *Rosa amblyotis* [20]
110		**L-Ascorbic acid** [Vitamin C]	C_6_H_8_O_6_	176.12		177.09	145.07			*Papaya*; *Strawberry* [51]; *Phoenix dactylifera* [27]; *Rosa rugosa*; *Rosa amblyotis* [20]
111	Organic acid	**Gluconic acid** [Gluconate; Dextronic acid; Maltonic acid; Glycogenic acid]	C_6_H_12_O_7_	196.16		197	178.14	132.13; 160.13		*Medicago truncatula* [76]; *Ribes meyeri* [11]
112	Amino acid	**L-Tryptophan** [Tryptophan; (S)-Tryptophan]	C_11_H_12_N_2_O_2_	204.23		205.11	188.08; 157.05; 143.03			*Camellia kucha* [57]; Rapeseed petals [28]; Huolisu Oral Liquid [46];
113	Omega-5 fatty acid	**Myristoleic acid** [Cis-9-Tetradecanoic acid]	C_14_H_26_O_2_	226.36		227.18	209.18	192.25; 139.13	122.23	*F. glaucescens*, *F. herrerae* [65]; *Ribes fragrans* [63]
114	Methylantyhrone	**Emodinanthranol** [Emodin anthrone; 1,3,8-Trihydroxy-6-methylanthrone]	C_15_H_12_O_4_	256.25		257.15	197.07; 117.33	187.05		*Ventilago denticulata* [77]
115	Ribonucleoside composite of adenine (purine)	**Adenosine**	C_10_H_13_N_5_O_4_	267.24		268.12	136.11; 122.14			Huolisu Oral Liquid [46]; *Rosa rugosa*; *Rosa amblyotis* [20]; *Lonicera japonica* [13]
116	Omega-3 fatty acid	**Linolenic acid** (Alpha-Linolenic acid; Linolenate)	C_18_H_30_O_2_	278.43		279.29	261.40; 205.07; 167.07; 149.08			Jatropha [64]; *Ribes dikuscha* [58]; *Rosa rugosa*; *Rosa amblyotis* [20]
117	Anthraquinone	**Physcion** [Physcion; Emodin-3-methyl ether; Parientin]	C_16_H_12_O_5_	284.26		285.35	257.34; 244.20; 227.08; 201.21	231.02		*Radix polygoni multiflori* [78]; *Ventilago denticulata* [77]
118	Oxylipin	**13-Trihydroxy-Octadecenoic acid [THODE]**	C_18_H_34_O_5_	330.46	329.12		229; 211; 171; 139	211; 183; 161; 143		*Bituminaria* [52]; *Rosa rugosa*; *Rosa amblyotis* [20]; *Phoenix dactylifera* [27]
119	Iridoid glucoside	**Harpagide**	C_15_H_24_O_10_	364.35		365.10	203.06; 185.05; 165.16; 135.13	185.08; 146.99		Honey [67]
120	Unsaturated fatty acid	**Pentacosenoic acid**	C_25_H_48_O_2_	380.65		381.25	335.35; 265.27; 230.19; 180.02	221.16; 146.21		*F. glaucescens*, *F. herrerae* [65]
121	Anabolic steroid	**Vebonol**	C_30_H_44_O_3_	452.67		453.31	435.27	336.20; 226.23	209.19	*Hylocereus polyrhizus* [79]; *Rhus coriaria* [15]
122	Product of chlorophyll degradation	**Pheophytin A**	C_55_H_74_N_4_O_5_	871.20		872.41	593.19	533.16	461.20	*Physalis peruviana* [80]
